# Preparation, structural characterization and health benefits of polysaccharides from *Rubus chingii* Hu: a review

**DOI:** 10.3389/fphar.2025.1592046

**Published:** 2025-05-09

**Authors:** Chao Shan, Yingjia Zhu, Haizheng Bi, Xingyu Wang, Jingyuan Wang, Meng Wang

**Affiliations:** ^1^ Heilongjiang University of Chinese Medicine, Key Laboratory of Basic and Application Research of Beiyao, Ministry of Education, Harbin, China; ^2^ Sanya Jiyang Fulongtang Traditional Chinese Medicine Clinic, Outpatient Department, Sanya, China

**Keywords:** Rubus chingii Hu, polysaccharides, extraction, purification, structural characteristics, health benefit, application

## Abstract

*Rubus chingii* Hu (*R*. *chingii*) is a valuable edible and medicinal plant. It contains a variety of surprising active compositions. For example, polysaccharides, phenols, terpenes, organic acids, benzenes, and flavonoids. Among them, polysaccharides, as macromolecular compounds, are receiving increasing attention from researchers. Polysaccharides obtained from *R*. *chingii* have anti-tumor, anti-colitis, immunomodulatory, anti-obesity, antioxidant and cytoprotective effects. In this paper, the extraction, purification, structural characteristics and health benefits of *R*. *chingii* polysaccharides were reviewed, which laid the knowledge foundation and provided the direction for future research. In addition, the structure-activity relationship of *R*. *chingii* polysaccharides was analyzed. Its actual and potential applications in the fields of food, pharmaceuticals, and cosmetics have also been discussed. This provides inspiration for the future research and development of its health products and functional foods. In summary, by reviewing the research on *R*. *chingii* polysaccharides in recent years, it will help researchers deepen their understanding of them and facilitate further research on *R*. *chingii* polysaccharides.

## 1 Introduction

**FIGURE 1:**
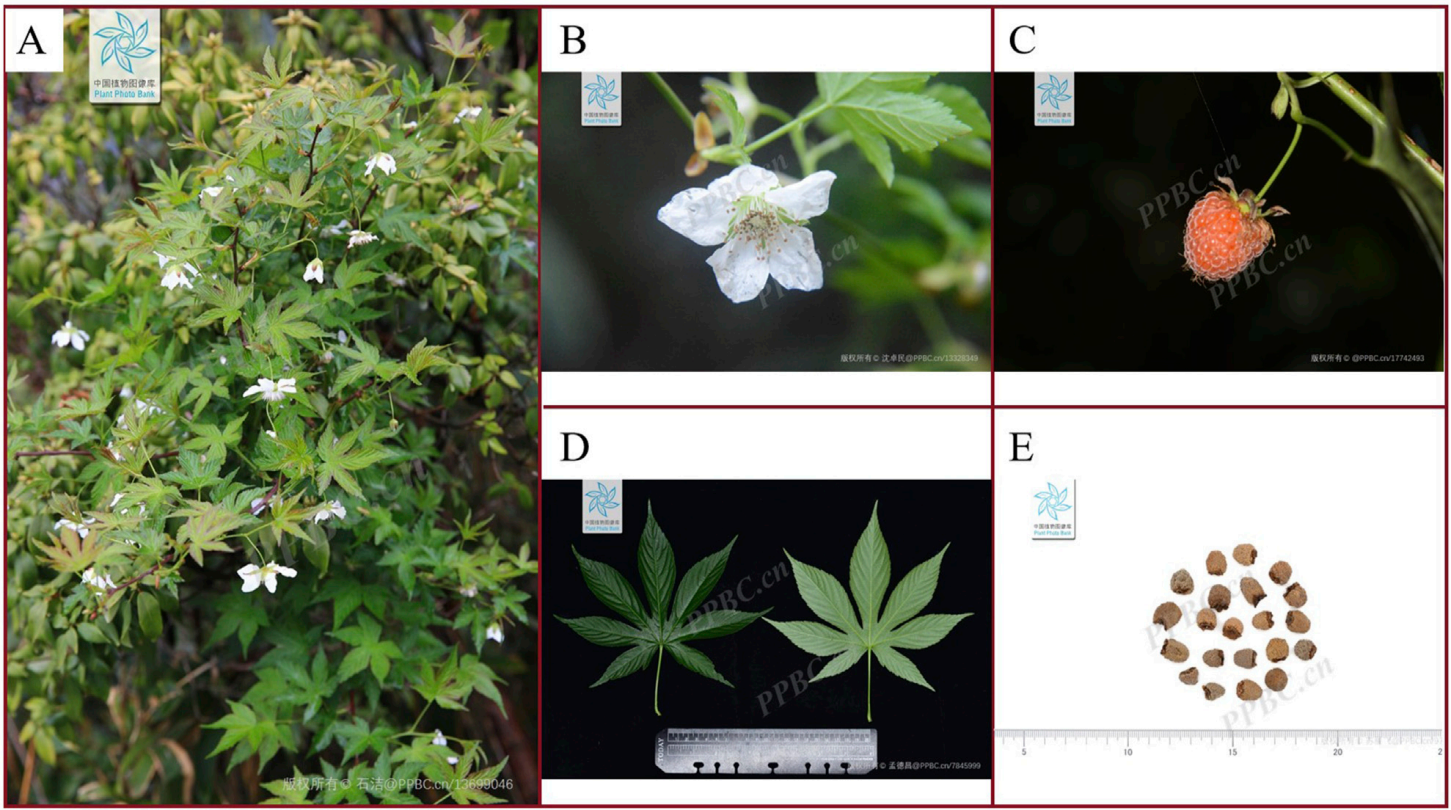
Plant morphology of *R. chingii* **(A)** A plant of *R. chingii.* **(B)** The flower of *R. chingii.* **(C)** The fruit of *R. chingii*. **(D)** The leaf of *R. chingii.* **(E)** The dried fruit of *R. chingii.* (*The plant images in this article are from Plant Photo Bank of China, PPBC).

*Rubus chingii* Hu (*R. chingii*), also known as Fu Penzi, is a rattan shrub of the genus *Rubus* in the Rosaceae family (Chen et al., 2025). Its plants are slender, ranging in height from 1.5 to 3 m, and are covered with many thorns. The leaves are nearly rounded, with a diameter of about 4–9 cm, and the base of the leaves is heart shaped. Its fruit is tightly clustered from many small drupes, forming a conical or flattened cone shape. The height of the fruit ranges from 0.6 to 1.3 cm, with a diameter of 0.5–1.2 cm (Wu M. et al., 2024). It usually blooms in March-April and bears fruit in May-June. The plant morphology of the *R. chingii* is shown in Figure 1. *R. chingii* is found in China and occasionally in Japan, where they grow at low to medium elevations and are commonly found on hillsides and roadside in the sun or shade of bushes (Hua et al., 2023). It has a history of approximately 1,500 years of consumption and medicinal use (Yu et al., 2019a). The ripe fruit of *R. chingii* has a delicious taste and is rich in nutrients such as vitamin A, vitamin C, calcium, potassium, magnesium, as well as a large amount of fiber. It can provide nutrition for the body and is very important for people’s daily lives (Chen et al., 2025). Besides, the ripe fruit of *R. chingii* can also be used for cooking, making various dishes such as salads, jams, desserts, beverages, etc. It is interesting that the unripe fruit of *R. chingii*, which changes from green to green-yellow, can also be used as a traditional Chinese medicine (TCM) (Wu et al., 2022). In 2012, the first batch of food and medicine homology released by the National Health and Family Planning Commission of the People’s Republic of China included *R. chingii* (He et al., 2023). The 2020 edition of the Chinese Pharmacopoeia also includes the use of *R. chingii*, which is mainly used to treat nocturnal emissions, enuresis, impotence, and premature ejaculation (He et al., 2020). Based on this, more and more consumers, nutritionists, and natural botanists are paying attention to *R. chingii*.

The fruits, leaves, and roots of *R. chingii* are important sources of active components (Han et al., 2012; Wang et al., 2023a). In recent years, a large number of research reports on *R. chingii* have discovered some important and effective small molecule component, including phenols, terpenes, organic acids, benzenes, and flavonoids (Li et al., 2021a; Wan et al., 2021; Wang et al., 2023b). With the deepening of research, polysaccharide has also been discovered as an active macromolecular component (Tian et al., 2024; Ghosh and Abdullah, 2024). The polysaccharides extracted from *R. chingii* have complex structures, diverse types, and satisfactory biological activities, such as anti-tumor, anti-colitis, immunomodulatory, anti-obesity, antioxidant, and cytoprotective effects (Xiong et al., 2024). These characteristics make it have broad application prospects in the development of functional foods and drugs (Tian et al., 2025; Sun et al., 2025). Existing reports indicate that polysaccharides have become important functional food ingredients due to their unique physicochemical properties and biological activities. The extensive biological activity of *R. chingii* polysaccharides deserves further research for use as additives in functional foods and food packaging materials. In addition, the therapeutic potential of *R. chingii* polysaccharides may also make them an important source of novel drugs or adjuvant therapies. In short, *R. chingii* has broad development space and potential.

Nowadays, people have a higher pursuit of health, and more and more people are paying attention to dietary therapy. Therefore, the demand for products with dual medicinal and edible values has greatly increased (Livingstone et al., 2023). *R. chingii* has attracted the attention of researchers due to its rich nutritional content and unique pharmacological activity. Based on this, there are many reviews about *R. chingii*. Although these reviews have made significant contributions, most of them focus on small molecules and lack a comprehensive summary of polysaccharides with important active compounds. The research and application of *R. chingii* polysaccharides are in a rapid development stage. However, there are still some shortcomings in the research of *R. chingii* polysaccharides. For example, the structure-activity relationship analysis of *R. chingii* polysaccharides mainly focuses on monosaccharide composition, molecular weight, and glycosidic bond types, while there is relatively less research on specific functional groups. Moreover, the quality control standards for *R. chingii* polysaccharides are not yet perfect. To address these issues, this article comprehensively integrates the extraction and purification, structural characteristics, health benefits, structure-activity relationship, and potential applications of *R. chingii* polysaccharides. By identifying research gaps and trends, provide ideas and references for future research on *R. chingii* polysaccharides.

## 2 Extraction and purification of *R. chingii* polysaccharides

The various uses of *R. chingii* are closely related to the chemical composition they contain. *R. chingii* contains various chemical components, but polysaccharides have gradually become an important direction in *R. chingii* research due to their unique biological activity, diverse structure, and low side effects. Summarizing the extraction and purification methods of *R. chingii* polysaccharides can lay a solid foundation for expanding the *R. chingii* industry chain and optimizing polysaccharide extraction process parameters.

### 2.1 Extraction

**FIGURE 2:**
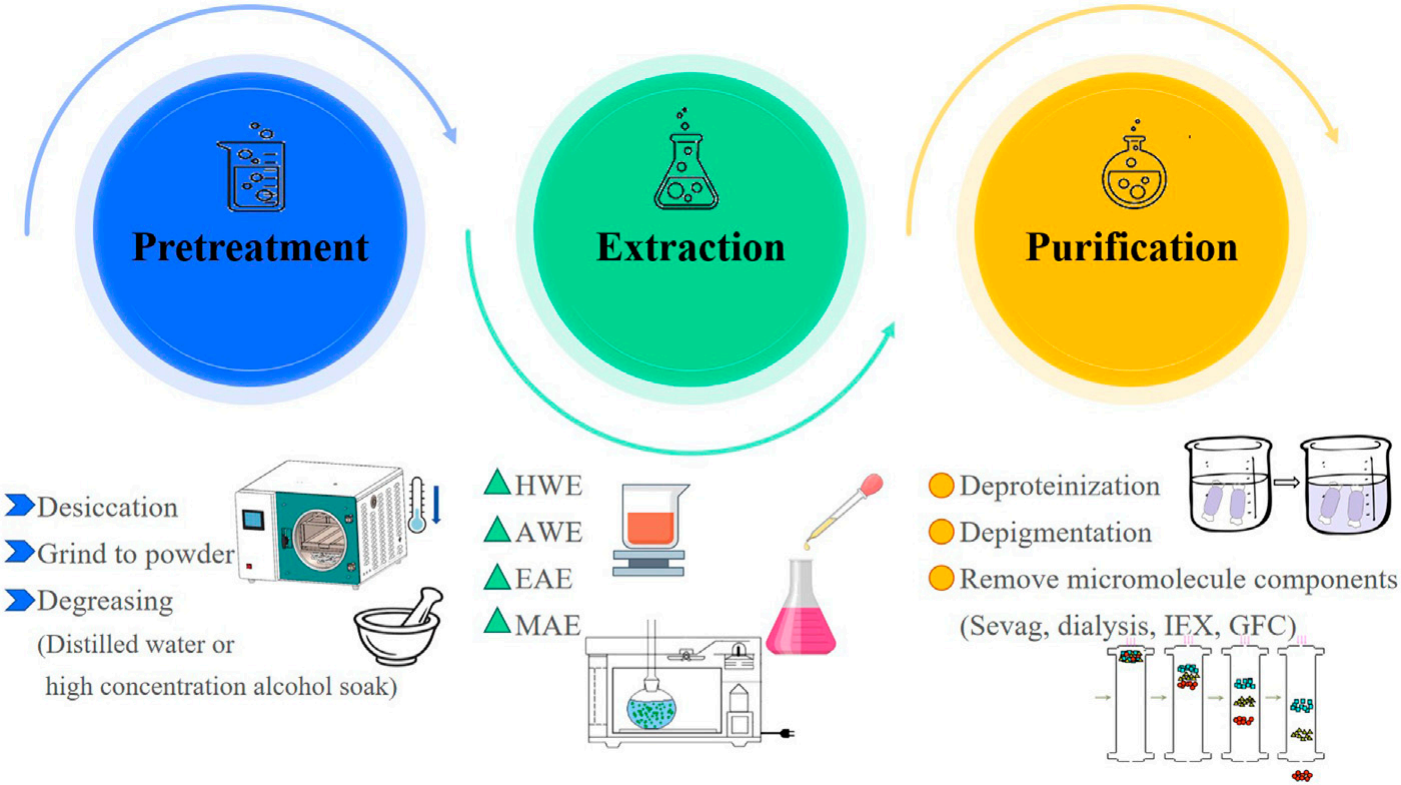
Extraction and purification process of *R. chingii* polysaccharide.

The extraction of *R. chingii* polysaccharides often uses water extraction method, which is widely used due to its simple operation, low cost, and green safety. With the increasing attention to polysaccharides, a series of complex extraction techniques have been developed and applied. For example, microwave-assisted extraction (MAE) and enzyme assisted extraction (EAE) (Li et al., 2021b; Yang et al., 2024). In this paper, the extraction methods of *R. chingii* polysaccharides were reviewed, and the relevant information about their extraction conditions was shown in Table 1 and Figure 2.

**TABLE 1:** Methods for extraction and purification of *R. chingii* polysaccharides.

Part	Name	Extraction					Purification		Ref
		Method	Time	Condition	Solid-liquid ratio	Yield	Name	Method	
Fruit	RCP	HWE	1 h	80°C	1: 20	NA	pRCP	Centrifuged, DEAE seplife FF, dialysis	Luo et al. (2023)
Fruit	F-Ps	HWE	2 h	100°C	1: 31	NA	F-Ps-1, F-Ps-2, F-Ps-3, F-Ps-4	DEAE-Sepharose fast flow column, DEAE cellulose-52	Zhang et al. (2015)
Leaf	L-Ps	HWE	3.09 h	87.92°C	1: 30.91	9.57%	L-Ps-1, L-Ps-2, L-Ps-3		
Fruit	RP	HWE	4 h	90°C	1: 10	1.67%	NA	Alcohol precipitation, dialyzed	Ke, Bao and Chen (2021)
Fruit	RP	HWE + MAE	2 h	50°C	1: 10	0.33%	NA	Alcohol precipitation	Ke, Bao and Chen (2019)
Fruit	RFPs	HWE + EAE	2 h	55°C	Cellulase: papain = 1: 1	3.62%	NA	Alcohol precipitation, dialyzed	Su et al. (2021)
NA	NA	HWE	4 h	80°C	1: 10	14.32%	RCHP-S	DEAE cellulose-52 column, Gel filler G-25 Sephadex, Sephacryl S300	Kong et al. (2022)
Unripe fruit pomaces	RP	Acid water extraction	1 h	80°C	1: 20	3.52% ± 0.76%	NA	Alcohol precipitation, dialyzed, centrifuged	Chen et al. (2022)
	URP	Ultrasonic modification	1 h	Ice-bath, 400 W	1: 25	3.52% ± 0.76%	NA	Dialyzed	

Abbreviation: HWE, hot water extraction; MAE = microwave-assisted extraction; EAE, enzyme assisted extraction; NA = not available.

An important step before extracting polysaccharides is to remove fat soluble substances from the sample, which is also known as sample pretreatment (Valladares-Diestra et al., 2022). The pretreatment method was one of the factors affecting the extraction rate of polysaccharides (Lee et al., 2015). Luo et al. (2023) pretreated *R. chingii* fruits using different methods, including immersion in distilled water, 50% ethanol, and immersion in 50% ethanol solution followed by ultrasonic treatment at a ratio of 1: 3 (w/v) for 30 min. Then, hot water extraction (HWE) was used to obtain polysaccharides, and the effects of three different pretreatment methods on the extraction rate were evaluated. The extraction rate of polysaccharides was the highest in the samples after ultrasonic assisted ethanol soaking. A three-step method was used to optimize the pretreatment conditions to improve the polysaccharide yield, namely, two-level Plackett-Burman design, Box-Behnken design (BBD) and three-dimensional response surface plot. The BBD model calculated the optimal values of the three factors, and the predicted extraction rate was 8.34%, which was equivalent to the actual extraction rate of 8.30% ± 0.07% (Luo et al., 2023).

The HWE method is the most used method for extracting polysaccharides from *R. chingii*, with extraction rates ranging from 0.33% to 14.32% (Zhang et al., 2015; Ke et al., 2021; Kong et al., 2022). The factors affecting the extraction rate include extraction temperature, extraction time and solid-liquid ratio. The method is suitable for large-scale industrial extraction, low cost and convenient operation (Chen et al., 2022). Zhang et al. (2015) obtained polysaccharides (L-Ps) from *R. chingii* leaves by using HWE, and optimized the extraction conditions of polysaccharides from *R. chingii* leaves by using response surface method. The optimal extraction conditions were as follows: extraction temperature was 87.92°C, extraction time was 3.09 h, solid-liquid ratio was 1: 30.91 g/mL and the extraction yield were 9.57% (Zhang et al., 2015). Ke et al. (2021) obtained polysaccharides (RP) from *R. chingii* by water extraction and alcohol precipitation, and the extraction rate was 1.67% (Ke et al., 2021).

Novel extraction methods such as MAE and EAE have also been applied to extract polysaccharides from *R. chingii*. These methods have their own characteristics, and suitable extraction techniques should be selected based on the parts extracted from plants and the characteristics of polysaccharides (Zhao et al., 2017; Ullah et al., 2019; Li et al., 2024). When a single extraction method could not meet the extraction requirements, a combination of two extraction methods was usually adopted to protect the polysaccharide structure and increase the polysaccharide yield (Wen et al., 2020; Kumar Jha and Nandan, 2022). The polysaccharide can be extracted from *R. chingii* fruit by MAE, which is extracted in hot water and then extracted by microwave (Ke, H., Bao, T., & Chen, W. 2019). Microwaves can make water molecules vibrate rapidly, thus generating heat, promoting the rupture of cell wall, and improving the solubility and extraction efficiency of polysaccharides (Mirzadeh et al., 2021). Su et al. (2021) obtained the crude polysaccharide extract by water extraction and alcohol precipitation, then mixed it with a complex enzyme (cellulase and papain, 1: 1) to further purify *R. chingii* polysaccharide (RFP), with a yield of 3.62% (Su et al., 2021). Studies have shown that in the process of polysaccharide extraction, the yield is closely related to the plant cell wall (Nai et al., 2021). Alkaline conditions will destroy the hydrogen bond between cellulose and hemicellulose in the plant cell wall, so as to release the polysaccharide (Wu et al., 2013). Citric acid can degrade the colloidal substances in the cell wall and dissolve the polysaccharide (Minjares-Fuentes et al., 2014). The enzyme solution was added to the extraction solution to use pectinase and cellulase to enzymize the pectin and cellulose in the cell wall, destroy the cell wall, dissolve the polysaccharide, and then increase the extraction rate.

Lastly, but equally important, when selecting an appropriate method for extracting polysaccharides from *R. chingii*, in addition to using extraction rate as an indicator, attention should also be paid to the impact of the extraction method on polysaccharide activity. The biological activity of *R. chingii* polysaccharides is the core of their application in various fields. Different experimental conditions can have some impact on the biological activity of *R. chingii* polysaccharides. Chen et al. (2022) compared the effects of two different extraction methods (HWE and ultrasound assisted extraction) on the antioxidant activity and *α*-glucosidase inhibitory activity of *R. chingii* polysaccharides. The results showed that both the *R. chingii* polysaccharide RP (obtained by HWE) and URP (obtained by ultrasound assisted extraction) had good antioxidant and *α*-glucosidase inhibitory activity. In addition, the antioxidant damage effect of URP is superior to RP, as it can effectively reduce H_2_O_2_ induced oxidative damage in human liver L02 cells by lowering intracellular reactive oxygen species (ROS) levels. Therefore, when choosing a suitable extraction method, comprehensive consideration should be given to effectively promote the development of extraction methods and maximize the role of *R. chingii* polysaccharides.

### 2.2 Purification

In order to obtain higher purity *R. chingii* polysaccharides for subsequent analysis and research, it is necessary to purify the extracted and preliminarily decontaminated polysaccharides (Shi L. 2016). The purification of *R. chingii* polysaccharides is a complex process involving multiple steps. Alcohol precipitation is a common purification method for polysaccharides, which uses different concentrations of ethanol or acetone to precipitate polysaccharides with different relative molecular weights (Guo et al., 2016). In addition, the crude polysaccharide contains proteins, pigments, inorganic salts, monosaccharides and other small molecular substances, which can be removed by ion exchange chromatography, gel filtration chromatography, macroporous adsorption resin, Sevag, dialysis (Tang et al., 2020; Zheng et al., 2023). Ion Exchange Chromatography (IEX) is a technique for separating ions and polar molecules based on their affinity differences to the ion exchanger. IEX plays an important role in the purification of polysaccharides and the commonly used ion exchangers include DEAE cellulose 52 column and DEAE FF cellulose column (Huang et al., 2022). Luo et al. (2023) loaded raspberry crude polysaccharide (RCP) onto a chromatography column containing DEAE seplife FF and eluted with NaCl solution to obtain higher purity polysaccharides. In addition, in some experiments, *R. chingii* polysaccharide eluted by ion exchange chromatography was further purified by gel filtration chromatography (G-25 Sephadex, polyacrylate S300, Superdex-200) to obtain refined *R. chingii* polysaccharide (Liu et al., 2018; Huang et al., 2022). Kong et al. (2022) purified *R. chingii* polysaccharide by DEAE cellose-52 column, and then further purified the obtained polysaccharide by gel column chromatography to obtain a new *R. chingii* pectin polysaccharide. In recent years, there has been great progress in the research of *R. chingii* polysaccharides. However, like other polysaccharides, the purification methods for *R. chingii* polysaccharides have developed slowly, mainly due to the variety of polysaccharides, complex structures, large molecular weights, and wide distribution.

## 3 Structural characteristics of *R. chingii* polysaccharides

**FIGURE 3:**
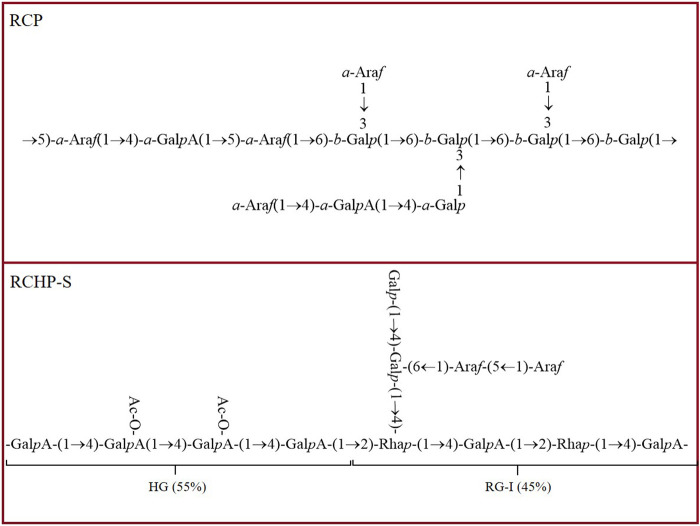
The chemical structure of *R. chingii* polysaccharides.

The structural characteristics of *R. chingii* polysaccharides have been studied and reported, including monosaccharide composition, molecular weight, side chain connection and main chain. Their structure is complex and diverse, which is inextricably linked to their remarkable biological activity. Table 2 provides comprehensive information on the sources, names, molecular weights, monosaccharide composition, chemical structure, analytical methods, and related references of *R. chingii* polysaccharides. Figure 3 shows the chemical structure of known possible *R. chingii* polysaccharides.

**TABLE 2:** Structural characteristics of *R. chingii* polysaccharides.

Name	Molecular weight	Monosaccharide composition	Chemical structure	Analytical method	Ref
pRCP	74.86 kDa	Fuc: Ara: Gal: Glc: Xyl: Man: GalA: GlcA = 1.91: 215.64: 213.84: 11.72: 8.32: 13.91: 46.44: 30.60	The main chain of pRCP consisted of → 3,6)-*β*-Gal*p* (1 → and → 5)-*α*-Ara*f* (1→, and its side chain was composed of *α*-Ara*f* (1 → linked to the C3 position of → 3,6)-*β*-Gal*p* (1 →	FT-IR, NMR, Methylation analysis, SEM	Luo et al. (2023)
L-Ps-1	17 kDa	Rha: Ara: Xyl: Glc: Gal = 2.47: 4.75: 4.12: 1: 2.48	Ps-1 was heteropolysaccharides with a glucan as backbone chain	UV, IR, GC, HP-GPC	Zhang et al. (2015)
F-Ps-3	81 kDa	Rha: Ara: Xyl: Glc: Gal = 4.21: 14.72: 1.63: 1: 3.22	F-Ps-3 was heteropolysaccharides with a glucan as backbone chain		
RP	703.9 kDa	GalA: Ara: Gal: Glc: Rha: Man: GlcA: Fuc = 39.78: 28.42: 8.78: 7.30: 3.90: 1.23: 1.00: 0.51	The backbone of RP consists of (1 → 5)-linked Ara*f*, (1 → 3,4)-linked Gal*p* and (1 → 3)-linked Gal*p*	SEC-MALLS-RI system, FT-IR, Methylation, GC-MS	Ke, H., Bao, T., & Chen, W. (2021)
RP	837.82 kDa	Man: Rha: GlcA: GalA: Glc: Gal: Ara: Fuc = 1.31: 4.41: 1.13: 43.20: 8.65: 9.51: 31.17: 0.61	RP mainly consists of galacturonic acid and arabinose with copious 1 → 2 glycosidic linkages in its backbone	HPLC, HPGPC, FT-IR, GC, Smith degradation	Ke, H., Bao, T., & Chen, W. (2019)
RFP	51.7 kDa	Man: Rha: GlcA: GalA: Glc: Gal: Ara: Fuc = 0.08: 0.17: 0.16: 0.48: 5.73: 1.00: 1 0.79: 0.03	NA	HPLC, FT-IR, GPC	Su et al. (2021)
RCHP-S	NA	Man: Rha: GlcA: GalA: Glc: Gal: Ara = 1.52: 19.08: 1.64: 41.98: 2.29: 20.61: 12.88	RCHP-S was composed of 1,4-Gal A and 1,2-Rha. It is mainly composed of HG-type pectin domains, and also contains a small amount of RG-I	HPGPC, HPLC, NMR, methylation	Kong et al. (2022)
RP	NA	Gal: Rha: Ara: Glc: Xyl: Man: GalA = 8.45: 2.39: 9.15: 13.65: 2.17: 1.65: 62.48	RP had a linear backbone of *α*-1, 4 linked GalA residues	HPGPC, NMR, SEM	Chen et al. (2022)
URP	NA	Gal: Rha: Ara: Glc: Xyl: Man: GalA = 8.14: 1.79: 7.08: 12.03: 1.62: 1.49: 67.76	NA		
RP2	180 kDa	Man: Rha: Glc: Gal: Ara: Fuc = 0.06: 0.33: 1.00: 0.08: 0.31: 0.15	NA	HPLC-DAD	Huang et al. (2024)

Abbreviations: glucose = Glc, galactose = Gal, rhamnose = Rha, arabinose = Ara, xylose = Xyl, mannose = Man, glucuronic acid = GlcA, galacturonic acid = GalA, fucose = Fuc, not available = NA.

### 3.1 Monosaccharide composition

High performance anion exchange chromatography (HPAEC), gas chromatography (GC) and high-performance liquid chromatography (HPLC) are common methods for the analysis of polysaccharide (Chen et al., 2025; Sripisut et al., 2024; Liu et al., 2025). Different raw materials, extraction methods and purification techniques have obvious effects on the composition of *R. chingii* polysaccharides. Previous studies have reported differences in the monosaccharide composition of *R. chingii* polysaccharides and their proportions.

Zhang et al. (2015) extracted polysaccharide L-Ps-1 from *R. chingii* leaves and analyzed its monosaccharide composition by acid hydrolysis and GC. The results showed that L-Ps-1 was mainly composed of five monosaccharides: rhamnose (Rha), arabinose (Ara), xylose (Xyl), glucose (Glc) and galactose (Gal), among which Xyl was the most abundant monosaccharides. F-Ps-3 is a polysaccharide derived from *R. chingii* fruit. Compared with L-Ps-1, it contains more Ara and less Xyl. The difference in monosaccharide composition of the two polysaccharides may be the reason for the difference in their biological activity (Zhang et al., 2015). RP2 is a homogeneous polysaccharide from *R. chingii* fruits. The monosaccharide composition of RP2 was determined by PMP pre-column derivatization and HPLC-DAD analysis. The analysis results show that RP2 is mainly composed of mannose (Man), Rha, Glc, Gal, Ara and fucose (Fuc), and the molar ratio is 0.06: 0.33: 1.00: 0.08: 0.31: 0.15 (Huang et al., 2024). There is a significant difference between the monosaccharide composition of L-Ps-1 and RP2, indicating that the source affected the type of polysaccharides. RP is a kind of pectin polysaccharide obtained from *R. chingii* by acid water extraction method, and the monosaccharide composition analysis shows that it is mainly composed of Gal, Rha, Ara, Glc, Xyl, Man and galacturonic acid (GalA) (Chen et al., 2022). RFPs is a water-soluble polysaccharide obtained from *R. chingii* by EAE. Its main monosaccharides include Man, Rha, GlcA, GalA, Glc, Gal, Ara and Fuc (Su et al., 2021). Compared to RP, RFPs has more Fuc and less Xyl. Therefore, the extraction technology affects the composition of polysaccharides.

### 3.2 Molecular weight

High-performance gel permeation chromatography (HPGPC), SEC-MALLS-RI system and high-performance size-exclusion chromatography (HPSEC) are commonly used to measure the average molecular weight (Mw) of polysaccharides (Gómez-Ordóñez et al., 2012). At present, the Mw of *R. chingii* polysaccharides reported is mostly between 17–837.82 kDa. The Mw of polysaccharides extracted from different parts was different. Zhang et al. (2015) purified two polysaccharides F-Ps-3 and L-Ps-1 from fruits and leaves, and established effective Mw information by HPGPC, which showed that their Mws were 81 kDa and 17 kDa, respectively (Zhang et al., 2015). pRCP and RP are polysaccharides from *R. chingii* fruits, but their Mw are quite different (74.86 kDa and 837.82 kDa) (Ke et al., 2019; Luo et al., 2023).

### 3.3 Chemical structure

Fourier transform infrared spectroscopy (FT-IR), ultraviolet-visible spectroscopy (UV-vis), nuclear magnetic resonance (NMR) and methylation analysis are commonly used to characterize the structure of polysaccharides (Wang et al., 2020; Feng et al., 2022). Each of these methods has its unique advantages and application range, which can deeply analyze the structural characteristics of polysaccharides from different angles. Methylation analysis focuses on the analysis of the branch structure and linking mode of polysaccharide, and through specific chemical modifications and subsequent analytical steps, the linking sequence and the location of branch points between each monosaccharide unit in polysaccharide molecules can be clearly presented. pRCP is an acidic heteropolysaccharide, and the types of glucoside bond were analyzed by methylation. The dominant types of glucoside linkage were t-Gal*p*, 3-Gal*p*, 4-Gal*p*,6-Gal*p* and 3,6-Gal*p*. Further analysis showed that pRCP consisted of → 3,6)-*β*-Gal*p* (1→and→5)-*α*-Ara*f* (1→ as the main chain, and the side chain was mainly composed of *α*-Ara*f* (1 → linked to the C3 position of → 3,6)-*β*-Gal*p* (1→ (Luo et al., 2023). Smith degradation refers to the decomposition of polysaccharides into small molecule polyols and undamaged polysaccharide fragments through steps such as periodate oxidation, borohydride reduction and acid hydrolysis, so as to infer the glycosidic bond connection mode and structural characteristics of polysaccharides. Smith degradation analysis showed that the type of glycosidic bond of RP is mainly 1→2 chain (Ke et al., 2019). NMR is one of the most authoritative and reliable techniques for the identification of polysaccharide structures, which can accurately determine the sequence of glycosidic bond, branching structure, monosaccharide composition and the configuration of sugar ring due to its high resolution and accurate resolution of molecular structure details. RCHP-S is a pectin polysaccharide with 1, 4-GalA and 1, 2-Rha as main chains. According to NMR and methylation analysis results, RCHP-S is a highly methylated mixed pectin, containing two domains of type HG and type RG-I (Kong et al., 2022). FT-IR can detect the characteristic absorption peaks of different functional groups in polysaccharide molecules, and quickly provide key information about the type of glycosidic bond and the configuration of sugar ring, which lays a foundation for the preliminary judgment of the structural framework of polysaccharides. RP is a polysaccharide from *R. chingii*. FT-IR and NMR analysis showed that its backbone was composed of (1→5) linked Ara*f*, (1→3, 4) linked Gal*p* and (1→3) linked Gal*p*. There are also relatively low content (1→2, 4) connected Gal*p*, end-connected Glc*p*, and (1→2) connected Glc*p* (Ke et al., 2021). In addition, the different extraction sites also affected the structure of polysaccharides. L-Ps-1 and F-Ps-3 are leaf and fruit polysaccharides from *R. chingii*, respectively, and both are heteropolysaccharides with glucan as the main chain (Zhang et al., 2015). The structure modification also changed the structure of polysaccharides. RP is also a pectin polysaccharide with a linear skeleton of *α*-1,4 attached to GalA residues. After ultrasonic modification, polysaccharide URP was obtained with higher HG content and lower RG-I content than RP. In addition, ultrasonic treatment changed the content of hydroxyl and GalA units but did not affect the structure of the pectin backbone (Chen et al., 2022). RP is a polysaccharide from *R. chingii*. FT-IR and NMR analysis showed that its backbone was composed of (1→5) linked Ara*f*, (1→3, 4) linked Gal*p* and (1→3) linked Gal*p*. There are also relatively low content (1→2, 4) connected Gal*p*, end-connected Glc*p*, and (1→2) connected Glc*p* (Ke et al., 2021). RCP is a functional polysaccharide with anti-obesity activity. Its main stem is →3,6)-*β*-Gal*p* (1→ and →5) -*α*-Ara*f* (1→), and the side chain is composed of *α*-Ara*f* (1→ C3 position →3,6)-*β*-Gal*p* (1→) (Wu M. et al., 2024). The structural characteristics of *R. chingii* polysaccharides were summarized and analyzed above, which provided valuable information for the structural study of *R. chingii* polysaccharides.

### 3.4 Morphological analysis

Scanning electron microscopy (SEM) is a common method for observing the surface microstructure and morphology of polysaccharides (Ngono et al., 2020; Yang et al., 2023). Characterization of the morphological surface of polysaccharides plays an important role in understanding the physicochemical properties and biological activities of polysaccharides (Chen et al., 2022). At present, there are few research on the micromorphology of *R. chingii* polysaccharides. The relevant information is summarized and analyzed as follows. The surface morphology of pRCP at different magnifications was observed, and the results showed that it had a smooth surface and a porous layered structure, suggesting that it has the potential to be used as an encapsulation material for drug delivery (Luo et al., 2023). Other studies have analyzed that RFP has rough irregular surface, random distribution of particles, and high polymerization (Su et al., 2021). RP is a pectin polysaccharide from immature fruit residue, which has a silky, smooth and delicate surface. The ultrasonic modified URP showed that the filamentous pectin was broken, and a large amount of spherical pectin was produced (Chen et al., 2022). This is due to the mechanical effect caused by ultrasound, which destroys the original structure (Jiang et al., 2023). In short, the characterization of micromorphology is of great significance for understanding the structure and function of polysaccharides, quality control, scientific research and application development.

## 4 Health benefits of *R. chingii* polysaccharides

**FIGURE 4:**
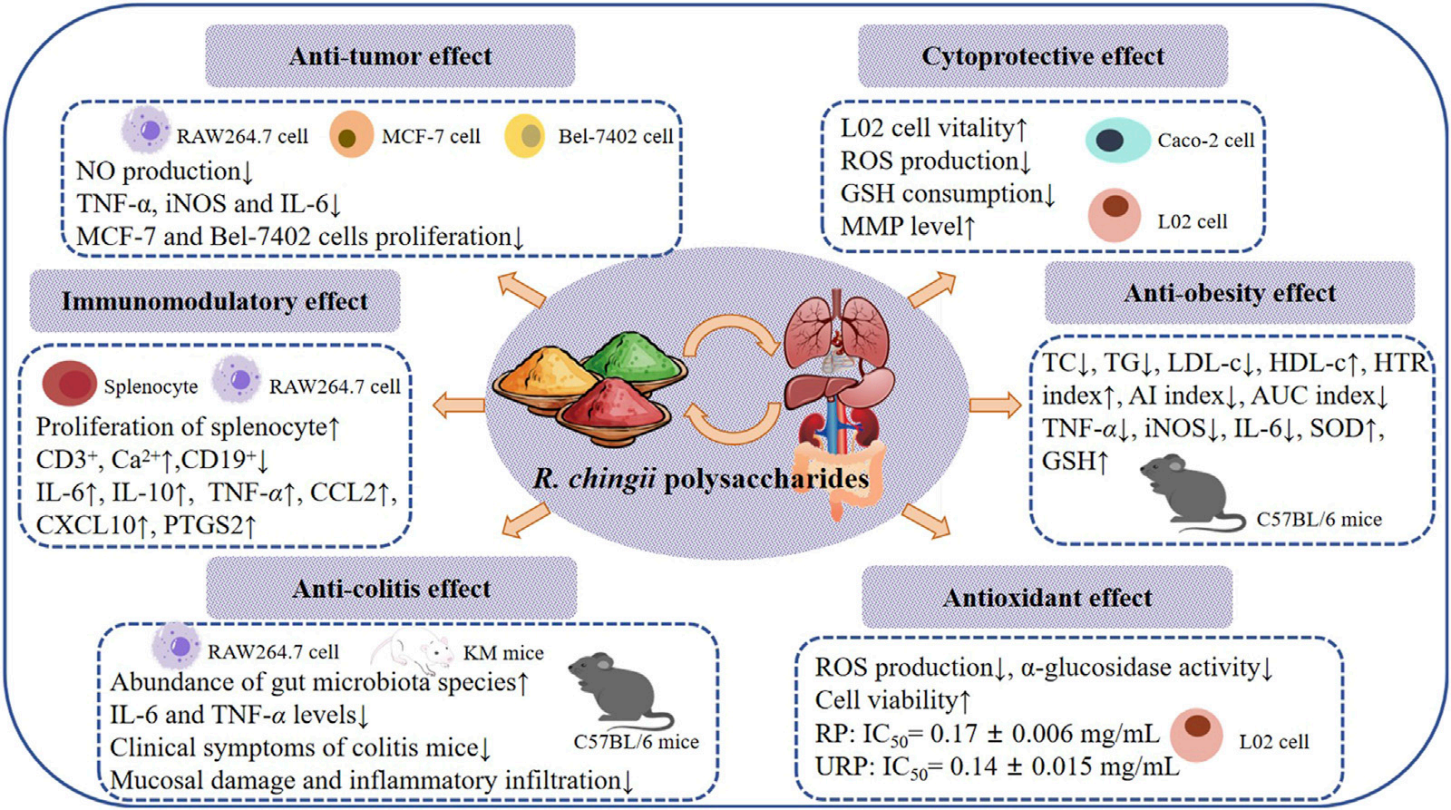
Health benefits of *R. chingii* polysaccharides.

The fruit of *R. chingii* is both edible and medicinal. It can treat impotence, premature ejaculation, enuresis, frequent urination and other diseases, the root can treat cough, toothache, nausea and vomiting and other diseases (Li et al., 2021). As a nutrient-rich fruit and traditional medicinal plant, *R. chingii* is widely used as a raw material for healthy food and nutritional supplement products in the food industry and medical and health fields (Zhang et al., 2017; Luo et al., 2024). Many literature have proved that polysaccharides extracted from *R. chingii* have a variety of potential important biological activities, and detailed information is shown in Table 3 and Figure 4.

**TABLE 3:** Health benefit and mechanism of *R. chingii* polysaccharides.

Biological activities	Polysaccharide name	*In vitro* or *in vivo*	Indicated concentrations	Models/test system	Action or mechanism	Ref
Anti-tumor effect	F-Ps and L-Ps	*In vitro*	0.125–2 mg/mL	RAW264.7 cell, MCF-7 cell, and Bel-7402 cell	NO production, TNF-α, iNOS, IL-6 mRNA expression and MCF-7 and Bel-7402 cell proliferation were inhibited	Zhang et al. (2015)
Anti-colitis effect	pRCP	*In vivo*	0.25 g/mL	C57BL/6 mice	Increased the abundance of gut microbiota species. Decreased the abundance of *Erysipelatoclostridium* and Negativibacillus	Luo et al. (2023)
	RCHP-S	*In vitro*	50, 100 and 200 μg/mL	RAW264.7 cell	Inhibition of IL-6 and TNF-α levels. Inhibited NO production	Kong et al. (2022)
		*In vivo*	200 and 50 mg/kg	KM mice	Alleviating the clinical symptoms of colitis mice. Relieve mucosal damage and inflammatory infiltration. Inhibit the secretion of TNF-α	
Immunomodulation effect	RFPs	*In vitro*	25, 50 and 100 μg/mL	Splenocyte	Promote the proliferation of splenocyte and release of NO. Upregulated CD3^+^, Ca^2+^ and downregulated CD19^+^	Su et al. (2021)
	RFPs	*In vitro*	12.5–200 μg/mL	RAW264.7 cell	Promote proliferation. Improved phagocytosis ability and pinocytosis activity of macrophages. Increase the secretion of ROS. Increase the production of IL-6, IL-10, TNF-α, CCL2, CXCL10, NO and PTGS2	Yu et al. (2019b)
Cytoprotective effect	RP	*In vitro*	5–250 μg/mL	L02 cell	Improve cell vitality. Inhibit ROS production. Reduce GSH consumption	Ke, H., Bao, T., & Chen, W. (2019)
	RP	*In vitro*	50, 100 and 150 μg/mL	Caco-2 cell	Inhibit ROS production and GSH consumption. Improve MMP level	Ke, H., Bao, T., & Chen, W. (2021)
Anti-obesity effect	RP2	*In vivo*	100 and 400 mg/kg	C57BL/6 mice	Reduce the levels of TC, TG and LDL-c. Increased HDL-c levels. Improve the HTR index. Reduce AI index and AUC index. Inhibition LPO levels. Increase SOD and GSH levels	Huang et al. (2024)
	RCP	*In vivo*	100 mg/kg	C57BL/6J mice	Decreased the rate of weight gain and liver to iWAT ratio. Reduce the levels of TC, TG and LDL-c. Improve glucose metabolism and insulin sensitivity. The mRNA expression levels of pro-inflammatory cytokines and pro-antioxidant indices were inhibited	Wu et al. (2024a)
Antioxidant effect	RP and URP	*In vitro*	0.025, 0.05 and 0.1 mg/mL	L02 cell	Inhibition of ROS production. The IC_50_ value of RP was 0.17 ± 0.006 mg/mL. The IC_50_ value of URP was 0.14 ± 0.015 mg/mL. Inhibition of *α*-glucosidase activity	Chen et al. (2022)

### 4.1 Anti-tumor effect of *R. chingii* polysaccharides

Polysaccharides obtained from natural plants have been shown to have good anti-tumor activity (Zhang et al., 2021). The anti-tumor activity of polysaccharide may be attributed to its immunomodulatory ability (Guo et al., 2022). Studies have shown that some pro-inflammatory cytokines can promote the penetration of immune cells into infected tissues, thus significantly improving the effectiveness of tumor therapy (Lei et al., 2023; Zhou et al., 2024). Zhang et al. (2015) extracted polysaccharide (F-Ps, L-Ps) from *R. chingii* fruits and leaves by HWE method and evaluated its effect on NO production in RAW264.7 macrophages. The results showed that 400 g/mL F-Ps and L-Ps had NO cytotoxicity and could inhibit the production of NO, with inhibition rates of 23.56% and 30.06%, respectively. RT-PCR results showed that F-Ps and L-Ps significantly inhibited the increased mRNA expression of TNF-*α*, iNOS and IL-6 in RAW264.7 cells induced by LPS. Among them, L-Ps had the strongest inhibitory effect on TNF-*α*, and mRNA expression decreased from 34.00 ± 1.23 to 21.19 ± 1.16. MTT assay is a common method to evaluate cell viability. The researchers used MTT method to observe the proliferation inhibition rate of breast cancer cell MCF-7 and cancer cell Bel-7402 with the concentration of 0.125–2 mg/mL F-Ps and L-Ps, and analyze their anti-tumor effect. The results showed that F-Ps and L-Ps inhibited the proliferation of both cancer cells, and the inhibition of MCF-7 was the best, and the activity of L-Ps was stronger than that of F-Ps. At a concentration of 2 mg/mL, L-Ps treatment for 72 h had a strong inhibitory effect on the growth of MCF-7 cells, with an inhibition rate of 66.30% ± 0.61%. The above information indicates that polysaccharides F-Ps and L-Ps can reduce the secretion of NO by inhibiting the expression of iNOS gene and can directly affect the proliferation of breast cancer cells MCF-7 and liver cancer cells Bel-7402 (Zhang et al., 2015).

### 4.2 Anti-colitis effect of *R. chingii* polysaccharides

Ulcerative colitis (UC) is a chronic, non-specific inflammatory bowel disease with clinical symptoms including diarrhea, abdominal pain, mucous, pus, blood stool, etc (Ordás et al., 2012). The disease is characterized by recurrent episodes and remission, which seriously affect the quality of life of patients, so it is important to develop safe and reliable drugs to treat colitis (Wangchuk et al., 2024). Kong et al. (2022) isolated RCHP-S from *R. chingii* and identified it as a pectin polysaccharide by NMR and methylation analysis. The use of 2 μg/mL LPS can increase the secretion of NO in RAW 264.7 cells. Pretreatment with RCHP-S at different concentrations could reduce the production of NO in cells. It has been reported that many inflammatory factors play an important role in the development of colitis, including TNF-*α*, IL-1β, IL-6 and IL-8. iNOS is an important functional enzyme in cells and plays an important role in the production of NO. The results showed that RCHP-S (50, 100 and 200 μg/mL) treatment group inhibited the expression of iNOS, IL-1β, IL-6 and TNF-*α* related genes in RAW 264.7 cells to varying degrees. ELISA results indicated that RCHP-S could inhibit IL-6 and TNF-*α* levels. In addition, a DSS induced colitis mouse model was established, and the therapeutic effect of RCHP-S (50 mg/kg and 200 mg/kg) on colitis was observed. Colitis mice showed coarse hair color, weight loss, loose and bloody stools, and the length of the colon was shorter than that of the normal group. RCHP-S can alleviate the clinical symptoms of colitis mice. Pathological observation showed that mucosal injury, basal inflammatory cell infiltration, crypt structure abnormality and epithelial villi defect occurred in the colon of the model group. In the low-dose RCHP-S group, some crypts were missing, while in the high-dose group, the colonic mucosa was largely intact, with epithelial and crypt morphology. ELISA results showed that RCHP-S inhibited the secretion of TNF-*α*. In summary, *R. chingii* polysaccharide can alleviate intestinal inflammatory response and play a certain role in the treatment of colitis (Kong et al., 2022).

An acidic heteropolysaccharide found in *R. chingii* fruit has also been shown to relieve high-fat diet (HFD)-induced colitis. The results showed that the number of gut microbiotas in HFD-fed mice was significantly lower than that in the normal group, and the abundance of gut microbiota species in HFD mice was significantly increased after administration of pRCP. Notably, pRCP reduced the abundance of harmful bacteria (*Erysipelatoclostridium* and *Negativibacillus*). Studies have shown that *Erysipelatoclostridium* is a key mediator in the induction of obesity in mice. Therefore, pRCP is expected to play a beneficial role as a prebiotic to regulate intestinal flora (Luo et al., 2023). Polysaccharide has shown great significance and potential in the treatment of UC, and its good therapeutic properties and safety make polysaccharide a promising drug for the treatment of UC (Niu et al., 2021).

### 4.3 Immunomodulatory effect of *R. chingii* polysaccharides

Immunomodulatory is one of the important biological activities of polysaccharides (Zhou and Li, 2024). Su et al. (2021) reported an extraction method of crude *R. chingii* fruit polysaccharides by water extraction and alcohol precipitation, and the polysaccharide RFPs was obtained by a complex enzyme combined with Sevag purification method. Spleen cells of BALB/c mice were used to study the immunomodulatory activity of RFPs. The results showed that RFPs could promote the proliferation of spleen cells, and the release of NO. Flow cytometry was used to analyze the immunomodulatory effects of RFPs on lymphocyte proliferation *in vitro*. This clearly indicates that RFPs can upregulate CD3^+^, Ca^2+^ and downregulate CD19^+^, indicating that RFPS can mediate splenic cell activation. RNA-Seq further elucidates the mechanism of RFPs regulation of immunity, and the results show that RFPs balances Th1/Th2/Th17 response patterns through MAPK, NF-*κ*B, Jak-STAT and calcium signaling pathways. The results show that the RFPs has good immunomodulatory activity, which can be used as a good immunomodulatory and healthcare product in the medical care industry (Su et al., 2021).

**FIGURE 5:**
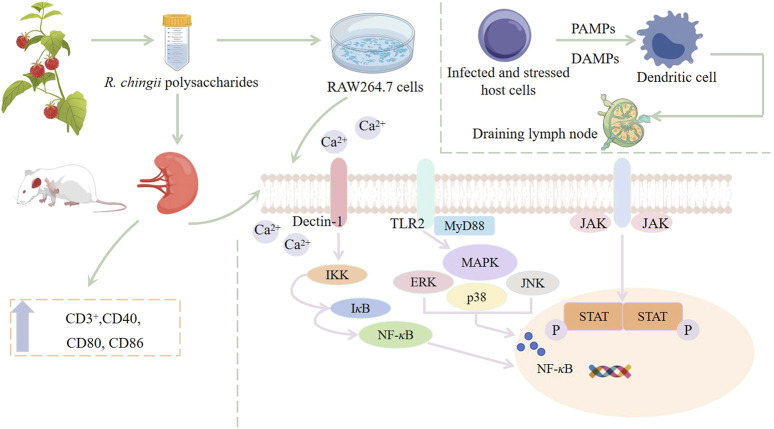
Potential mechanism of immunomodulatory effect of *R. chingii* polysaccharides.

The immunomodulatory activity of RFPs has been studied to influence the immune system by regulating macrophages. Macrophages are known as the “scavenger” of immune cells, which have a variety of functions, including presenting antigens, phagocytosis of foreign invaders, and secreting regulatory cytokines (Mehla and Singh, 2019; Yang et al., 2020). Xu et al. (2021) used MTT method to analyze the effect of RFPs on the viability of macrophages, and the results showed that 12.5–200 μg/mL of RFPs could promote the proliferation of RAW 264.7 cells. Phagocytosis of macrophages plays a key role in the induction and regulation of specific immune responses. The results of neutral red and FITC-dextran indicated that RFPs could improve phagocytosis and pinocytosis activity of macrophages. Reactive oxygen species (ROS) play an important role in immunomodulatory and is the mediator of phagocytosis, antigen presentation, activation, cell lysis and differentiation (Zhao et al., 2022). Studies have shown that RFPs can increase the secretion of ROS in macrophages and the expression of surface molecules, such as CD40, CD80, CD86, MHC-I, and MHC-II. In addition, RT-qPCR analysis showed that RFPs increased the production of cytokines (IL-6, IL-10 and TNF-*α*), chemokines (CCL2 and CXCL10), NO and PTGS2 in macrophages, which was similar to the results of ELISA detection. In addition, the experiment also investigated which receptors RFPs regulate macrophage activation by acting on. The results showed that TLR2 blocking antibodies can significantly inhibit the production of TNF-*α*. It is speculated that TLR2 is involved in the early recognition and function of RFPs. The immunomodulatory mechanism of RFPs was analyzed using Western blotting, and the results were consistent with previous findings. RFPs mainly regulate macrophage immune activity through MAPK, NF-*κ*B, and Jak-STAT pathways (Xu et al., 2021) (Figure 5). The above preliminary exploration of the immunomodulatory activity of *R. chingii* polysaccharides has determined the mechanism, but there may be cross regulation between multiple signaling pathways activated by polysaccharides, and further research is needed to investigate the interactions between these pathways (Li et al., 2022).

### 4.4 Cytoprotective effect of *R. chingii* polysaccharides

Cells are the basis of organism growth and development, and also the basis of disease. The disappearance of normal cell structure and abnormal function will inevitably lead to disease, so protecting diseased cells is also one of the ways to protect the body from foreign objects (Juan-García et al., 2021). A large number of basic and clinical studies have shown that polysaccharides in natural plants have beneficial effects on cell protection (Chen et al., 2016; Premarathna et al., 2024). Palmitic acid (PA) can cause lipotoxicity of normal human hepatocyte cell line L02, which leads to liver and its complications. A polysaccharide RP was isolated from *R. chingii* fruit by hot water extraction, deproteinization and dialysis, which can protect L02 cells from palmitic acid damage. MTT assay showed that RP in the concentration range of 5–250 μg/mL had no obvious cytotoxicity to L02 cells, and RP in the concentration of 150 μg/mL had the greatest effect on cell viability (73.64% ± 4.25%). Lipotoxicity induced by PA is closely related to oxidative stress (Yu et al., 2019b). DCFH-DA probe detection showed that RP inhibited ROS production in a dose-dependent manner. Previous studies have reported that PA induction significantly reduces intracellular glutathione (GSH). Compared with the blank group, the PA processing group decreased to 50.00% ± 7.12%, while the RP processing group significantly reduced the consumption of GSH. GSH regulates ROS levels, cleans up free radicals and is an important antioxidant. Therefore, RP can protect PA-induced L02 cytotoxicity by affecting GSH. These results provide a new research direction for the full development and utilization of RP for human health (Ke, Bao and Chen, 2019).

To further verify the cytoprotective effect of RP, its effect on ethyl carbamate (EC) induced cytotoxicity was studied. MTT test results showed that the cell viability of RP treatment group was higher than that of normal group, and the highest cell viability was 99.80% ± 8.14% at the concentration of 150 μg/mL. Mitochondria, as the energy factories of cells, are involved in many important physiological processes (Zhang et al., 2022). EC-induced Caco-2 cells showed reduced MMP levels, which were mitigated by RP. In addition, the fluorescence experiment confirmed that the RP can play the role of cell protection by inhibiting the production of ROS and the consumption of GSH (Ke et al., 2021).

### 4.5 Anti-obesity effect of *R. chingii* polysaccharides

Huang et al. (2024) used DEAE-FF cellulose column to obtain three components (RP1, RP2 and RP3) by elution with sodium chloride, and confirmed that RP2 is a homogeneous polysaccharide that can play an anti-obesity role by affecting intestinal flora. Mice fed HFD were used as obesity models and treated with different concentrations of RP2. Compared with the model group, the levels of total cholesterol (TC), triglyceride (TG) and low-density lipoprotein cholesterol (LDL-c) in the RP2 treatment group were decreased, while the levels of high-density lipoprotein cholesterol (HDL-c) were increased. Furthermore, RP2 increases HTR value and decreases the atherogenic index (AI) and AUC index. Studies have shown that obesity affects the health of the liver. Histopathologic staining showed that the liver surface of mice in the model group was dark yellow and granular, with lipid deposition, vacuole, balloon degeneration and inflammatory infiltration, indicating that HFD induced severe liver injury and liver fibrosis. RP2 treatment can alleviate liver damage, with high doses being the most effective. LPO, SOD and GSH are associated with liver injury. ELISA results showed that RP2 could inhibit the increase of LPO induced by HFD and increase the decrease of SOD and GSH induced by HFD. Further studies showed that RP2 inhibits the NF-*κ*B classical inflammatory signaling pathway. Numerous studies have shown that gut microbiota plays a key role in regulating the interaction between HFD intake and the onset of obesity (Kang et al., 2022). Changes in the composition of intestinal flora may affect the body’s energy homeostasis, systemic inflammation, lipid metabolism and insulin sensitivity (Cai et al., 2022). HFD can cause damage to the intestinal barrier leading to chronic inflammation and microbial imbalance (Frazier et al., 2022). HE staining showed that severe inflammatory invasion of colon, intestinal villi shedding and intestinal gland atrophy were significantly improved in model group compared with high-dose RP2 treatment group. *In vitro* experiments have shown that RP2 effectively inhibits LPS-induced TJ protein loss and inflammatory response in Caco-2 cells. The 16s rRNA method is a common method to evaluate the variety and abundance of microbial groups in the gut (Matsuo et al., 2021). The results show that RP2 can enrich the gut with beneficial bacteria including *Lactobacillus*, *Bifidobacterium*, *Desulfovibrio*, and *Turicibacter* (Huang et al., 2024). Therefore, *R. chingii* polysaccharide has a potential as a prebiotic preparation to improve gut health and metabolic function with a view to alleviating obesity and metabolic syndrome.

Obesity can cause liver inflammation, hyperglycemia, hyperlipidemia, endotoxemia and oxidative stress (Kaplan et al., 2016; Chai et al., 2022). Wu L. et al. (2024) obtained polysaccharide components (RCP) from *R. chingii* and investigated the mitigative effects of PCR on HFD-induced hepatitis and oxidative stress. Compared with HFD-fed mice, RCP intervention significantly reduced the rate of weight gain and liver to iWAT ratio and improved the ability to metabolize sugar and insulin sensitivity. The results show that RCP can reverse the increase of TC, TG and LDL-c levels and the decrease of HDL-c levels caused by HFD. RT-PCR assay detected mRNA expression levels of pro-inflammatory cytokines and pro-antioxidant indicators inhibited by RCP to play a role in the treatment of inflammation and oxidative damage in the liver of obese mice. In addition, RCP increased the abundance of *Dubosiella*, which promotes butyrate production, thereby protecting the intestinal barrier and reducing circulating LPS levels in the liver of obese mice (Wu L. et al., 2024). In conclusion, *R. chingii* polysaccharides have anti-obesity effects and reduce inflammation and oxidative stress induced by obesity.

### 4.6 Antioxidant effect of *R. chingii* polysaccharides

ROS play an important role in the body, participate in normal physiological processes, and also play a key role in the occurrence of diseases (Zeng et al., 2015; Forrester et al., 2018). Their production and clearance in the cell are dynamic balance process, and the right amount of ROS plays an important role in cell signaling and defense mechanisms, but excessive ROS can lead to oxidative stress and damage to cells and tissues (Liu J. et al., 2022; Jomova et al., 2023). *R. chingii* polysaccharide (RP) and ultrasonic modified polysaccharide URP have good antioxidant activity. MTT results showed that RP and URP had no significant cytotoxicity to human liver L02 cells at concentrations below 0.1 mg/mL. H_2_O_2_ can induce ROS production in human liver L02 cells and then cause oxidative damage to the cells. The cell viability test showed that RP could increase the viability of L02 cells by 78.52% and 83.39% at 0.05 mg/mL and 0.1 mg/mL concentrations. URP at 0.025 mg/mL, 0.05 mg/mL and 0.1 mg/mL could increase cell viability by 79.89%, 83.48% and 92.35%. In addition, RP and URP significantly inhibited ROS production in L02 cells at 0.05 mg/mL and 0.1 mg/mL concentrations, respectively. In addition, RP and URP have good DPPH scavenging ability, ABTS scavenging ability and FRAP ability, among which the effect on DPPH is the best, URP’s clear ability is stronger than RP. RP and URP at different concentrations inhibited *α*-glucosidase activity. The results showed that the IC_50_ value of RP was 0.17 ± 0.006 mg/mL, while the IC_50_ value of URP was 0.14 ± 0.015 mg/mL (Chen et al., 2022). In conclusion, antioxidant activity is an important biological activity, which is of great significance for maintaining body health and food stability. With the deepening of research, the evaluation and application of antioxidant activity will play a greater role in many field.

## 5 Structure-activity relationship of *R. chingii* polysaccharides

The study of structure-activity relationship of polysaccharides is helpful to reveal the relationship between polysaccharide structure and biological activity, which has important guiding significance for the development of new polysaccharide drugs (Lin and Huang, 2022). The relationship between Mw and polysaccharide activity is an important aspect in the study of the structure-activity relationship of polysaccharides. In a certain range, polysaccharides with large Mw may have more complex spatial structure due to their more monosaccharides, various sugar bonding modes and polymerization types, which may improve their biological activity (Fernandes and Coimbra, 2023). L-Ps and F-Ps are two monosaccharides from *R. chingii*, both of which have anti-tumor activity, and the therapeutic effect of L-Ps is stronger than that of F-Ps through experiments. This may be attributed to the difference in the structure of polysaccharides. According to the structural analysis, both polysaccharides were composed of Rha: Ara: Xyl: Glc: Gal, and the molar ratio was 2.47: 4.75: 4.12: 1: 2.48 and 4.21: 14.72: 1.63: 1: 3.22, respectively. In addition, the molecular weights of L-Ps and F-Ps are 17 kDa and 81 kDa, respectively (Zhang et al., 2015). In summary, the Mw of polysaccharides is one of the important factors affecting their biological activity, and different Mw ranges may correspond to different biological activity performances. Appropriate Mw regulation can improve the biological activity and application value of polysaccharides (Zhang J. et al., 2023). The influence of monosaccharide composition on polysaccharide activity is a key factor in the study of structure-activity relationship of polysaccharides (Ferreira et al., 2015). RCP and RP2 are two polysaccharides from *R. chingii* fruits and have anti-obesity activities, but their monosaccharides are different. The monosaccharide composition of RCP is Fuc: Ara: Gal: Glc: Xyl: Man: GalA: GlcA, while RP2 is mainly composed of Man: Rha: Glc: Gal: Ara: Fuc (Luo et al., 2023; Huang et al., 2024). In addition, the composition of monosaccharides is the same, the proportion of different will also affect the biological activity of polysaccharides. RFP and RP are mainly composed of Man: Rha: GlcA: GalA: Glc: Gal: Ara: Fuc composition, molar ratio was 0.08: 0.17: 0.16: 0.48: 5.73: 1.00: 1.79: 0.03 and 1.31: 4.41: 1.13: 43.20: 8.65: 9.51: 31.17: 0.61. *In vitro* experiments show that RFP has immunomodulatory activity, and RP plays a good role in cell protection (Ke et al., 2019; Su et al., 2021). The main chain structure of polysaccharide is the basis of its biological activity, which has a direct influence on the activity of polysaccharide. The main chain of pRCP is composed of →3,6)-*β*-Gal*p* (1→ and →5)-*α*-Ara*f* (1→), while RCHP-S is a pectin polysaccharide and mainly composed of 1, 4-GalA and 1, 2-Rha. However, both pRCP and RCHP-S have been shown to have anti-colitis effects (Ke et al., 2021; Luo et al., 2023).

Structural modification of polysaccharides is an important means to improve their biological activity and application range (Deng et al., 2020). The physicochemical properties, bioactivity or pharmacokinetic properties of polysaccharides can be improved by changing some functional groups or structural characteristics of polysaccharides by chemical, physical or biological methods (Shah et al., 2024; Ziliani et al., 2024). Common structural modification methods of polysaccharides include sulfation, phosphorylation, acetylation, alkylation, sulfonation, carboxymethylation, etc. Ultrasonic modification is an effective method for the structural modification of polysaccharides. Chen et al. (2022) obtained a pectin polysaccharide (RP) from unripe *R. chingii* pomace and dissolved it in distilled water for ice bath ultrasound. The modified pectin polysaccharide (URP) was obtained by dialysis of the solution after ultrasound. The researchers evaluated the antioxidant activity of the two polysaccharides and the results showed that the antioxidant effect of URP was stronger than that of RP. This shows that ultrasonic modification can improve the original biological activity of polysaccharide (Chen et al., 2022).

With the advancement of technology, a study has analyzed the structure-activity relationship of *R. chingii* polysaccharides using interpretable artificial neural network model. This is a method different from previous polysaccharide structure-activity relationship analysis, which not only improves research efficiency and prediction accuracy, but also brings new ideas and references to the field of polysaccharide research. This study used an artificial neural network (ANN) model for prediction and employed a gradient-weighted class activation mapping algorithm to explain the structure-activity relationship of *R. chingii* polysaccharides. The results showed that Mw, Ara, Gal, Glc, GalA content, and the sugar linkage patterns of → 3) - Ara*p* - (1 →, Ara*f* - (1 →, and → 4) - Gal*p* - (1 →) are the main factors affecting the immune enhancing activity of *R. chingii* polysaccharides (Lu et al., 2024). In summary, the structure of *R. chingii* polysaccharides is the key to clarifying the structure-activity relationship of polysaccharides. Detailed and in-depth structural studies can provide a more reliable scientific basis for the study of the structure-activity relationship of *R. chingii* polysaccharides. In the future, research on polysaccharide structure-activity relationship models will place greater emphasis on interdisciplinary integration and innovation. With the development and progress of big data technology and artificial intelligence, more advanced algorithms will be applied to the study of *R. chingii* polysaccharide structure-activity relationships.

## 6 Potential application of *R. chingii* polysaccharides

**FIGURE 6:**
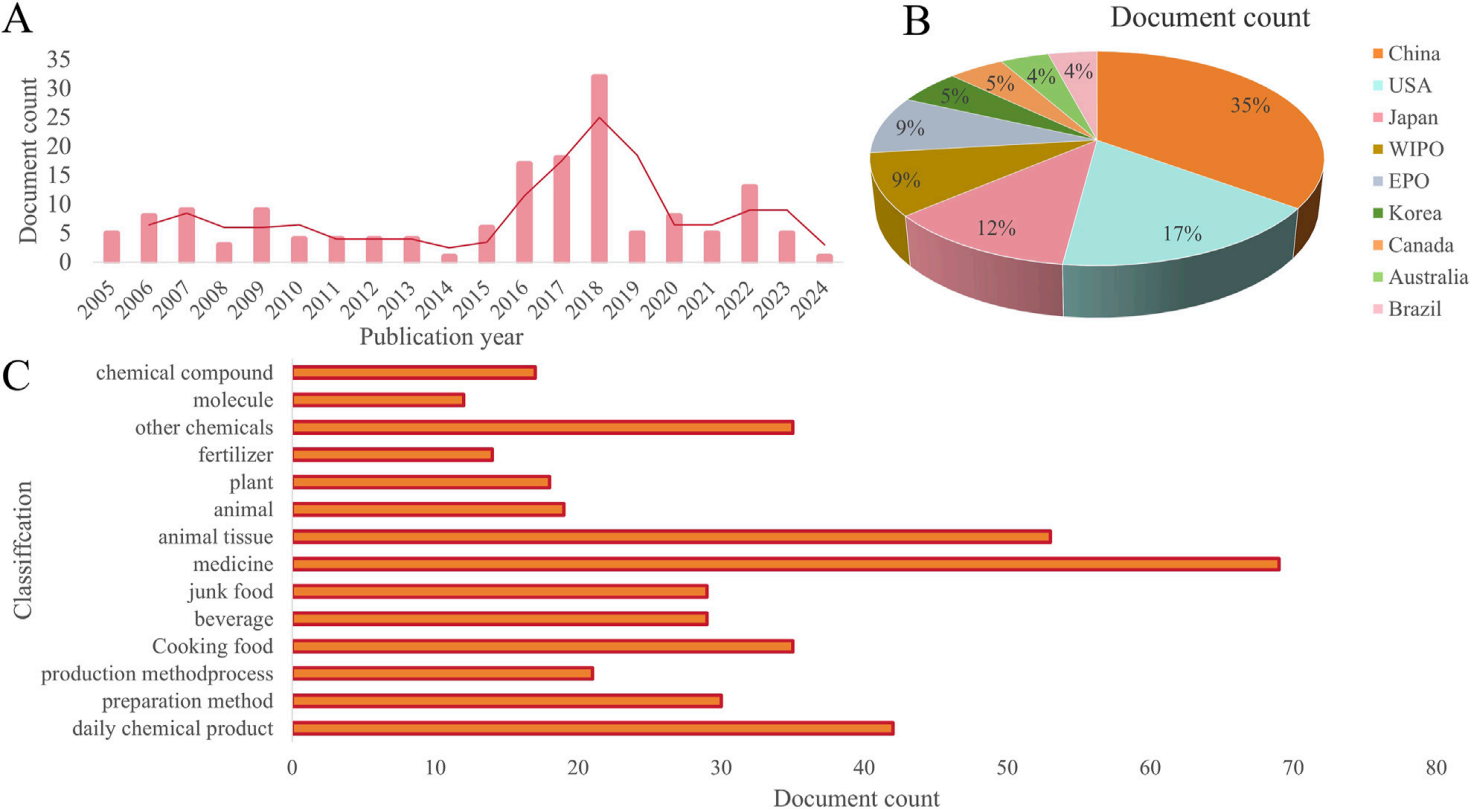
Current situation of patent inventions related to *R. chingi* polysaccharides **(A)** Patent applications per year. **(B)** Jurisdiction **(C)** Central product classification. (Source: https://t.incopat.com).

Polysaccharide is a kind of substance which comes from a wide range of sources, has the advantages of structural diversity, biocompatibility, stability, adhesion, degradability and so on, and widely exists in nature (Kumar et al., 2023; Sun et al., 2024). They not only play an important structural and energy storage function in the body, but also have a wide range of applications in the food industry, medicine, cosmetics and other fields because of their unique physical and chemical properties and biological activities (Rao et al., 2024). The current patent status of *R. chingii* polysaccharides in the world is shown in Figure 6. As you can see from the picture, there are many countries that apply for the patent for *R. chingii* polysaccharide, and the number of Chinese patents is 35%. The United States and Japan have similar percentages of patents, at 17% and 12% respectively. Korea, Canada, Australia and Brazil accounted for a smaller 5%, 5%, 4% and 4% of the total number of patents. In short, the research and development of *R. chingii* polysaccharide related products are still in its infancy. In the food industry, *R. chingii* polysaccharides are used as thickeners and emulsifiers in food products to improve taste and increase stability. Jelly, yogurt, fermented milk and other foods are often eaten by consumers, and *R. chingii* polysaccharide is used to improve the stickiness of food. Due to its special physicochemical properties, polysaccharides are also used as packaging materials and functional additives and stabilizers in the food industry. *R. chingii* polysaccharides have a variety of biological activities, such as antioxidant and regulation of gut flora, so they are also used in the development of functional and health foods. In the field of medicine, polysaccharide is a potential bioactive ingredient, which can be used not only for the preparation of drug materials, but also as a drug carrier for controlling the release of drugs. According to the existing studies, *R. chingii* polysaccharide has anti-tumor, anti-colitis, anti-obesity and other effects. A Chinese herbal composition with *R. chingii* as the main raw material has been prepared to treat or improve the symptoms of urinary incontinence, urinary frequency and nocturia. In addition, a composition for the treatment of gout is prepared by *R. chingii* and other TCM, each raw material component has a synergistic effect, the raw materials are natural animal and plant sources, the effect is mild, non-toxic side effects, can be taken for a long time, the therapeutic effect is significant, the efficiency is high, has the dual effect of prevention and treatment. In the cosmetics industry, polysaccharides have a good moisturizing effect, can increase the moisture content of the skin, improve the nutritional supply of the skin and the excretion of metabolic waste, is widely used in skin care products. Furthermore, the antioxidant effect is an important activity of polysaccharides, which has the potential to be used in facial masks and sunscreen products. In general, *R. chingii* polysaccharide has a variety of beneficial characteristics to the body, but the development and utilization of related products is still relatively small. In order to give full play to the potential application of *R. chingii* polysaccharide, it is necessary to enrich the types of *R. chingii* polysaccharide, strengthen the study of its structure-activity relationship, and further develop and promote functional food.

## 7 Conclusion and prospect

*R. chingii* is a kind of plant with the same origin as medicine and food, and has a long history of edible and medicinal use (Zhou et al., 2021). At present, polysaccharide macromolecular components have become a hot research hotspot because of their good biocompatibility, low toxicity and low side effects (Roman Maldonado et al., 2024; Zhang et al., 2024). Polysaccharides obtained from *R. chingii* have various physical properties, complex chemical structures, rich biological activities and wide applications. *In vivo* and *in vitro* experiments have confirmed that *R. chingii* polysaccharide has anti-tumor, anti-colitis, immunomodulatory, anti-obesity and cytoprotective effects on the body. In addition, *R. chingii* polysaccharides can also be used as thickeners, emulsifiers, antioxidants are widely used in food, pharmaceuticals and cosmetics.

*R. chingii* polysaccharide has an important development prospect in medicine, food and other fields, but different extraction methods and structural analysis techniques affect the physicochemical characteristics and chemical structure of polysaccharides. According to the literature, the extraction methods of *R. chingii* polysaccharide include hot water extraction, acid water extraction, microwave assisted extraction and enzyme assisted extraction. Each extraction of technology has its own advantages and disadvantages. The suitable extraction method should be selected according to the characteristics of polysaccharide. As we all know, extraction methods affect the molecular weight, monosaccharide composition and chemical structure of polysaccharides. Therefore, some new extraction technologies can be selected for obtaining *R. chingii* polysaccharides, such as ultrasonic assisted extraction, supercritical CO_2_ extraction, and low eutectic solvent extraction. Ultrasonic assisted extraction technology is a new high-efficiency separation and extraction technology, which can destroy cell structure and release polysaccharide through mechanical vibration and cavitation effect generated by ultrasonic waves (Zhang W. et al., 2023). This method is simple, safe, time-saving, widely applicable and low energy consumption. Supercritical CO_2_ extraction technology is a method of substance extraction using CO_2_ in supercritical state as a solvent. It has the advantages of strong extraction ability, high extraction efficiency, little loss of volatile components or damage to physiological active substances, and no solvent residue. The low eutectic solvent extraction method is one of the new methods in recent years. It uses a new medium and can dissolve a variety of substances through the combination of hydrogen bonds. This method has the advantages of low cost, easy synthesis, high solubility, non-toxicity and less damage to polysaccharide structure. The analysis of the chemical structure of polysaccharides is also a hot research topic. Up to now, the commonly used methods for structural analysis of *R. chingii* polysaccharides include GC, FT-IR, NMR, HPLC, HPGPC, etc. The above methods are mostly used to characterize the primary structure of polysaccharides, such as molecular weight, monosaccharide composition and glycosidic bond composition, while the higher structure of polysaccharides is rarely characterized. This indicates that the analytical methods of polysaccharides are not perfect enough, and the development of advanced structural characterization techniques of polysaccharides should be paid attention to in the future. The present study shows that atomic force microscopy (AFM) can provide the topological structure and spatial distribution information of polysaccharide molecules, and is suitable for analyzing the spatial structure of polysaccharide. X-ray diffraction (XRD) can obtain the crystal structure and molecular configuration information of polysaccharide (Liu Y. et al., 2022). Circular dichroism (CD) can be used to analyze the spatial structure and conformational transformation of polysaccharide and can be used to analyze the three-dimensional structure of polysaccharide.

*R. chingii* polysaccharide has a variety of surprising biological activities, among which the anti-tumor effect has received more and more attention. *R. chingii* polysaccharide can significantly inhibit the proliferation of tumor cells by affecting the expression of iNOS gene. Due to the low toxicity and obvious therapeutic effect of polysaccharide, it has a bright prospect as a raw material for the development of new anti-tumor drugs or adjuvant tumor drugs. Obesity is a common health problem that not only affects people’s appearance and quality of life, but is also associated with multiple health risks and diseases. *R. chingii* polysaccharide has a good resistance to inflammation caused by obesity, and it is the best treatment for colitis and hepatitis. At the same time, obesity can also lead to intestinal flora disorders, causing a series of gastrointestinal diseases. *R. chingii* polysaccharide can precisely play a beneficial role as a prebiotic to regulate intestinal flora. These results suggest that polysaccharide can be used as a good active ingredient for weight loss products and functional products for intestinal protection. In addition, antioxidant effects are one of the important health benefits of *R. chingii* polysaccharides. Studies have found that it can inhibit the production of a variety of oxygen free radicals, which makes it can be added to daily chemical products as an antioxidant, so that the body can delay aging and keep the skin hydrated. Safety, green and health has always been the goal pursued by the majority of consumers, the development and sales of related products not only promote the progress of science and technology but also promote the development of the economy.

This paper summarized the relevant literature of *R. chingii* polysaccharide, showed its unique physical properties and health benefits, provided the knowledge base and guidance direction for the future research of *R. chingii* polysaccharide, and provided enlightenment for solving the problem of the single extraction method and the backward structural analysis technology. It is hoped that more attention will be paid to *R. chingii* polysaccharides in the future, and more kinds of products that are beneficial to the body will be developed.

## Abbreviations

Ara, Arabinose; BBD, Box-Behnken design; EAE, Enzyme-assisted extraction; EC, Ethyl carbamate; FT-IR, Fourier transform infrared spectroscopy; Fuc, Fucose; Gal, Galactose; GalA, Galacturonic acid; GC, Gas chromatography; Glc, Glucose; HFD, High-fat diet; HPAEC, High performance anion exchange chromatography; HPGPC, High-performance gel permeation chromatography; HPLC, High-performance liquid chromatography; HWE, Hot water extraction; Man, Mannose; MW, Molecular weight; MAE, Microwave-assisted extraction; NMR, Nuclear magnetic resonance; IEX, Ion Exchange Chromatography; PA, Palmitic acid; ROS, Reactive oxygen species; Rha, Rhamnose; *R. chingii, Rubus chingii* Hu; SEM, Scanning electron microscopy; TCM, Traditional Chinese medicine; UC, Ulcerative Colitis; UV-vis, Ultraviolet-visible spectroscopy; Xyl, Xylose.
